# Nano-Confinement Effects on Structural Development and Organic Solvent-Induced Swelling of Ultrathin Carbon Molecular Sieve Films

**DOI:** 10.1021/acsami.1c03392

**Published:** 2021-04-28

**Authors:** Wojciech Ogieglo, Kepeng Song, Cailing Chen, Qiong Lei, Yu Han, Ingo Pinnau

**Affiliations:** † Functional Polymer Membranes Group, Advanced Membranes and Porous Materials Center, Division of Physical Sciences and Engineering, King Abdullah University of Science and Technology, 23955 Thuwal, Saudi Arabia; ‡ Nanostructured Functional Materials, Advanced Membranes and Porous Materials Center, Division of Physical Sciences and Engineering, King Abdullah University of Science and Technology, 23955 Thuwal, Saudi Arabia

**Keywords:** carbon molecular sieves, membrane technology, thin films, nanoconfinement, swelling, molecular separations

## Abstract

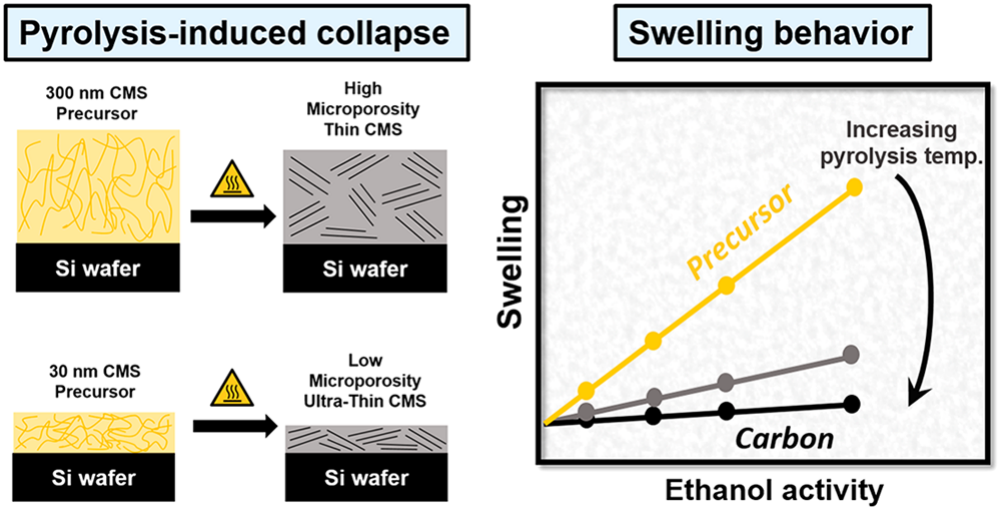

Successful implementation of carbon molecular sieve (CMS) membranes in large scale chemical processes inevitably relies on fabrication of high performance integrally skinned asymmetric or thin-film composite membranes. In principle, to maximize separation efficiency the selective CMS layer should be as thin as possible which requires its lateral confinement to a supporting structure. In this work, we studied pyrolysis-induced structural development as well as ethanol vapor-induced swelling of ultrathin CMS films made from a highly aromatic polyimide of an intrinsic microporosity (PIM–PI) precursor. Utilization of a light polarization-sensitive technique, spectroscopic ellipsometry, allowed for the identification of an internal orientation within the turbostratic amorphous CMS structure driven by the laterally constraining support. Our results indicated a significant thickness dependence both in the extent of pyrolytic collapse and response to organic vapor penetrant. Thinner, substrate-confined films (∼30 nm) collapsed more extensively leading to a reduction of microporosity in comparison to their thicker (∼300 nm) as well as self-supported (∼70 μm) counterparts. The reduced microporosity in the thinner films induced changes in the balance between penetrant-induced dilation (swelling) and filling of micropores. In comparison to thicker films, the initial lower microporosity of the thinner films was accompanied by slightly enhanced organic vapor-induced swelling. The presented results are anticipated to generate the fundamental knowledge necessary to design optimized ultrathin CMS membranes. In particular, our results reinforce previous findings that excessive reduction of the selective layer thickness in amorphous microporous materials (such as PIMs or CMS) beyond several hundred nanometers may not be optimal for maximizing their fluid transport performance.

## 1 Introduction

Carbon molecular sieve (CMS) membranes represent a special class of inorganic amorphous membrane materials with a significant potential in technologically important molecular separations such as hydrogen purification, natural gas processing, air separations, or carbon capture.^1−5^ Recently, CMS membranes have been demonstrated to be very effective in challenging organic solvent^6^ and olefin/paraffin^7−9^ separations. Consequently, CMS membranes are perceived as potential future contributors in the necessary transition of our society toward more energy efficient industrial separations. The widespread use of this technology, however, relies on transformation of the CMS materials into high-performance thin-film membranes with highly selective and simultaneously highly efficient (high permeance) molecular transport. The most common strategy to achieve high permeances relies on a reduction of the selective layer thickness, typically to submicron range, to minimize transport resistance.

The significant potential of CMS thin-film composite membranes originates from their microporous nature. CMS materials possess a turbostratic structure, which can be approximated by considering graphene-like nanosheets randomly arranged in an amorphous network. The microporosity of the CMS materials is thought to have a bimodal character with smaller ultramicropores (<0.7 nm) and larger micropores (<2 nm) present simultaneously.^4^ This bimodal pore size distribution is responsible for outstanding molecular separation properties where ultramicropores provide high selectivities by a molecular sieving mechanism whereas the larger micropores are responsible for lowering overall transport resistance leading to high gas permeance. Similar to CMS materials, polymers of intrinsic microporosity (PIMs) typically contain a bimodal pore size distribution where highly contorted backbones trap large amounts of excess free volume in the solid state.^10^ The presence of micropores results in both PIMs and CMS outperforming the state-of-the-art polymeric membrane materials, sometimes by a very large margin.^11^ In addition, CMS membranes are highly chemically and thermally stable even in long-term field operation.^12,13^ However, similarly to other high free volume amorphous membrane materials, CMS membranes suffer from a progressive structural densification (physical aging) leading to significant reductions of permeance.^14,15^ Moreover, physical aging, and to some degree dilation (or swelling) induced by condensable gases like CO_2_ or organic vapors, are well-known to amplify in thinner films, particularly in the submicron region.^16−19^

In glassy polymers, including PIMs, the combination of nanoconfinement in the form of ultrathin films and the presence of penetrants is known to have significant consequences to their molecular separation performance.^19−23^ In particular, swelling resulting from interaction with organics which may be present in the feed mixtures^24^ has been shown to depend on film thickness. This effect originates from a growing influence of the interfaces on the overall behavior of thin films.^20,25^ While the topic of swelling in ultrathin organic polymer films has been addressed in the past 20 years, hardly any data exist on swelling of thin and ultrathin CMS films. Understanding of the solvent-induced swelling is indispensable to advance the important emerging applications of the CMS membranes, such as organic solvent reverse osmosis (OSRO) or organic solvent forward osmosis (OSFO).^1,26−28^

In this work, we investigated organic-vapor-induced swelling of supported CMS thin films produced by pyrolyzing a polyimide of intrinsic microporosity (PIM–PI) precursor in two thickness ranges: ∼300 and ∼30 nm. Combination of ex situ and in situ interference-enhanced spectroscopic ellipsometry allowed the swelling and refractive index changes to be resolved independently and provided strong evidence for a much larger relative pyrolysis-induced collapse in thinner ∼30 nm films as compared with thicker ∼300 nm films. The utilization of uniaxial anisotropic optical modeling indicated a large degree of structural orientation within the CMS films in the plane of the substrate driven by the laterally constraining substrate. Ethanol-induced swelling has been shown to strongly reduce with increasing pyrolysis temperature. At the same time, the balance between the fraction of microporosity accessible to the penetrant and the penetrant-induced swelling showed a subtle thickness dependence.

## 2 Experimental Section

### 2.1 Sample Preparation and Characterization

**Figure 1:**
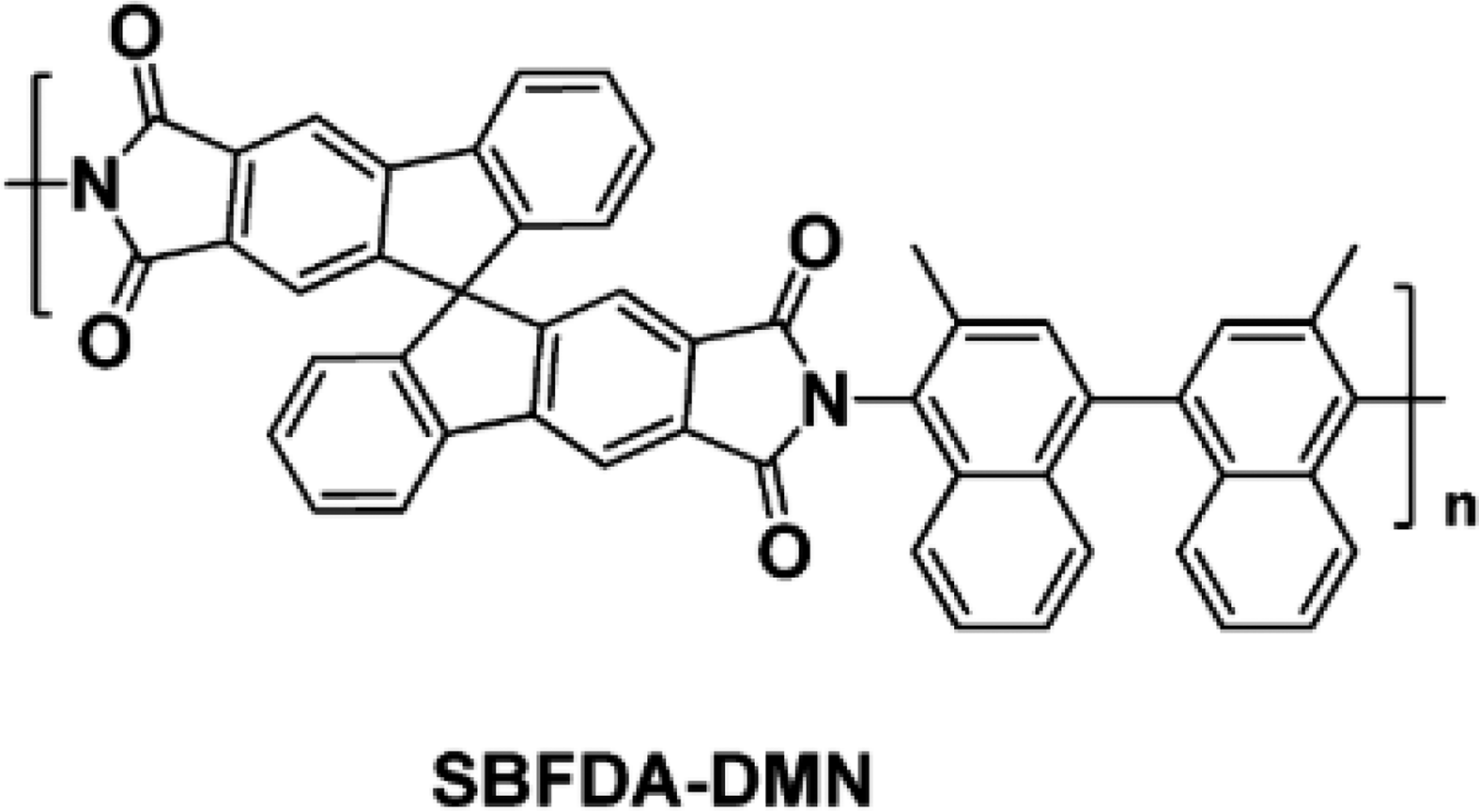
SBFDA-DMN polyimide with intrinsic microporosity (PIM–PI) used as carbon molecular sieve precursor in this study.

The CMS precursor, a polyimide of intrinsic microporosity, SBFDA-DMN (Figure 1), was synthesized by a polymerization reaction of spirobifluorene-based dianhydride with 3,3′-dimethylnaphthidine, as described previously.^29^ The polymer was characterized by a molecular weight *M*_n_ = 6.5 × 10^4^ g mol^–1^ with a polydispersity of 1.92 and an internal surface area of *S*_BET_ = 686 m^2^ g^–1^ measured at 77 K using N_2_. The thermal decomposition onset was determined by TGA at ∼520 °C (Supporting Information, Figure S1). The polymer combined a PIM character (high internal surface area and a rigid backbone) with a very high aromatic carbon content (84 wt %) which in our earlier work was proven to result in high-performance carbon molecular sieve membranes following pyrolysis.^15^

Thin SBFDA-DMN precursor films in two thicknesses, ∼300 and ∼30 nm, were spin-coated on top of 500 nm thermal silicon oxide wafer pieces (Si-Mat, Germany) from a chloroform solution of an adjusted concentration (between 0.5 and 3 wt %) at 2000 rpm. All silicon wafer pieces were separately characterized prior to film deposition to determine the exact silicon oxide thickness, which varied between about 493 and 498 nm. After spin-coating all samples were annealed at 50 °C for 16 h in air to remove the solvent. The pyrolysis process was conducted in a horizontal quartz tube furnace (Carbolite) under 1000 (STP) cm^3^ min^–1^ nitrogen flow at a ramp rate of 3 °C min^–1^ and 60 min dwell time at each temperature set point. Subsequently, the oven was left to passively cool within about 7–9 h. The pyrolysis set points were 500, 600, 700, and 800 °C, respectively. The oxygen concentration was monitored at the outlet of the furnace and was always below 5 ppm.

Transmission electron microscopy was performed with a low-base Titan electron microscope without Cs-corrector. The Cs was 1.2 mm, and the images were taken at high voltage (300 kV). The samples were cut with focused ion beam at low voltage. Raman spectra were recorded using 633 nm HeNe laser as excitation source on a Horiba Aramis device.

### 2.2 Ellipsometry Measurements and Data Analysis

**Figure 2:**
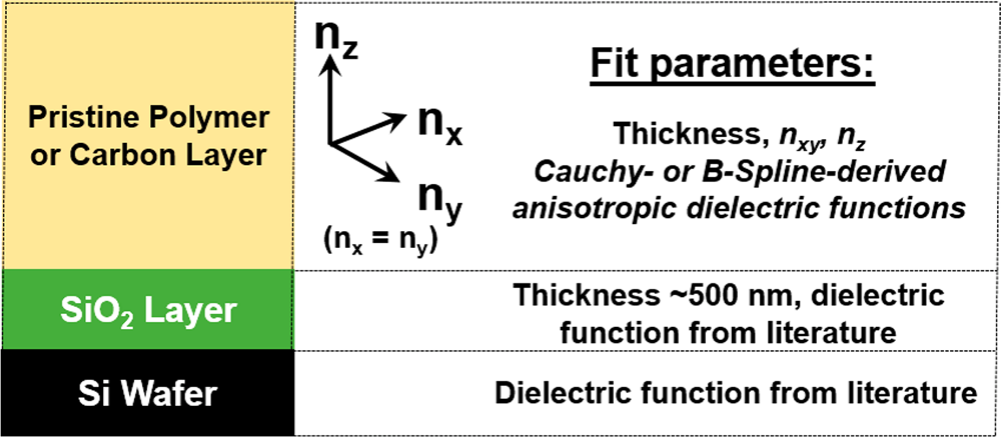
Scheme of the anisotropic, uniaxial optical model used for the sample analysis throughout this study.

A spectroscopic ellipsometer (M-2000 DI, J. A. Woollam, Co.) operating in a wavelength range of 193–1690 nm together with accompanying optical modeling software CompleteEASE v. 6.51 was employed throughout this study to conduct both ex situ and in situ (ethanol swelling) sample characterization. Ex situ analysis for both the thicker ∼300 and thinner ∼30 nm films was performed in a wavelength range of 600–1690 nm at a minimum of three angles of incidence (65, 70, and 75°), whereas 75° was used for in situ ethanol vapor experiments. Even though CMS are considered amorphous materials, structural orientation may develop upon confinement to substrates.^30^ To study the development of structural orientation within the CMS films as a result of pyrolysis and confinement to the substrate, we have chosen to employ anisotropic optical modeling. To preserve consistency, all samples were modeled using exactly the same, anisotropic (uniaxial), model. For the weakly light-absorbing samples (pristine precursor, 500 and 600 °C) a Cauchy-type model with fitted extinction coefficient was used. For the strongly light-absorbing samples (700 and 800 °C) a Kramers–Kronig consistent B-Spline model^31^ with a node resolution of 0.3 eV (for both *xy* and *z* components) was employed, Figure 2. To simplify the model by reducing the number of fit parameters only a difference in the zeroth order of the anisotropic *n*_*xy*_ and *n*_*z*_ component (“A” parameter) was fit. This procedure kept the real parts of the optical dispersion parallel, while the light absorption was fitted individually for each sample but was assumed the same in both *x*/*y* and *z* directions. The refractive index components were reported at 1000 nm, instead of at 632.8 nm as typically done, to avoid being too close to the edge of the modeling range (600–1690 nm). The presence of the ∼500 nm thick silicon oxide underneath each polymer/carbon film amplified the accuracy of the analysis versus the more broadly used native oxide wafers, as thoroughly investigated before.^15,32,33^ An example of the optical model is presented in the Supporting Information (Figure S2). Error bars were estimated based on the fit parameter uniqueness analysis assuming as threshold a 25% reduction of the mean squared error (MSE).

### 2.3 In Situ Ethanol Swelling

The in situ ethanol vapor swelling experiments were performed on freshly made CMS films in a commercially available (J. A. Woollam, Co.) flow cell at 21 ± 0.5 °C using a fully automated vapor generation system on freshly pyrolyzed samples (within 1 day from pyrolysis) by following exactly the same protocol for thick (∼300 nm) and thin (∼30 nm) samples. The desired ethanol vapor concentration was adjusted by two computer-controlled mass flow controllers (Alicat), one for the control of the pure carrier gas (nitrogen) and one for the control of ethanol-saturated stream (at 21 ± 0.5 °C). Both mass flow controllers always operated at a combined flow of 50 cm^3^ (STP) min^–1^. The protocol involved stabilization of the sample under pure carrier gas flow for 90 min and a subsequent stepwise exposure to increasing ethanol *p*/*p*_sat_ up to 0.8. Each step lasted 90 min. In situ ellipsometry data were continuously recorded every 5 s. An example of the in situ data is shown in the Supporting Information, Figure S3.

### 2.4 Approximate Calculation of Ethanol Concentration within the Pristine PIM–PI and Carbon Films

Ellipsometry-derived changes in thickness and refractive index can be used to approximately calculate the concentration of ethanol sorbed into the thin PIM–PI precursor and carbon films. In this work, the Clausius-Mossotti^34^ approach was used where the refractive index of the component *i*, *n*_*i*_, is related with the molar refraction, *R*_*j*_, molecular weight, *M*_*i*_, and density, ρ_*i*_, by
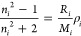
1The ratio *R*_*i*_/*M*_*i*_ is constant for a given substance and is denoted as *q*_*i*_. In a mixture of two components *i* and *j*, the refractive index *n*_mix_ carries contributions from all components. These contributions are additive and lead to the following expression containing concentrations expressed in units of density (g cm^–3^):
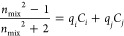
2In swollen films, the concentration of polymer (or carbon) reduces with swelling and can be calculated using the swollen and dry thicknesses as
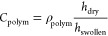
3Polymer and carbon densities were assumed to be the same as in self-standing thick films, as determined by Hazazi et al.^11^ and ranged from 1.13 for the pristine PIM–PI to 1.18 g cm^–3^ for the CMS prepared at 800 °C. In this study, the extremely high glass transition temperature of the precursor and carbon films as well as moderate ethanol-induced swelling assured that the Clausius-Mossotti-derived concentrations remained relatively accurate,^35^ in particular for films pyrolyzed above 500 °C.

## 3 Results and Discussion

**Figure 3:**
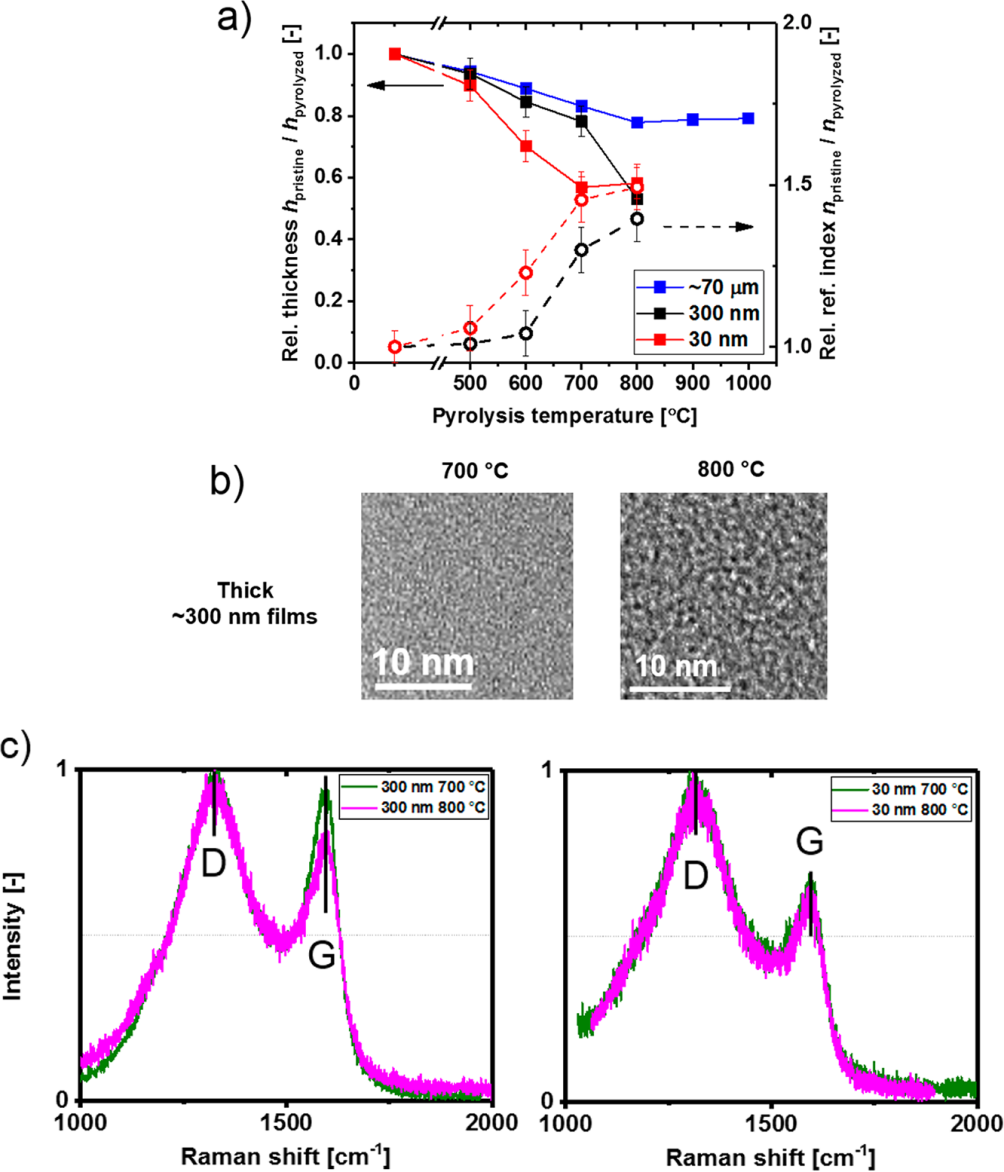
(a) Relative thickness and relative refractive index as a function of the pyrolysis temperature for both thick (∼300 nm) and thin (∼30 nm) polymer precursor films; ∼70 μm data is for a self-supported “bulk” film^15^ reprinted with permission from ref (15). Copyright 2019 American Chemical Society. (b) Cross-sectional transmission electron microscopy images showing the microstructure of the thick CMS films; (c) normalized Raman spectra (633 nm excitation wavelength) showing the typical “D” and “*G*” peaks observed in CMS materials for both ∼300 and ∼30 nm films.

The pyrolysis-induced thickness reduction of the CMS precursor films in both thick (∼300 nm) and thin (∼30 nm) films, is depicted in Figure 3a. The relative refractive index values were derived from the anisotropic model using an average refractive index calculated as *n* = (2*n*_*xy*_ + *n*_*z*_)/3 to reflect the relative changes in the overall film optical density^30^ and compare between the thick and thin films. We note here that in spectroscopic ellipsometry modeling thickness and refractive index data are to a large extent independent from each other and thus represent independent pieces of information about the properties of the pyrolyzed films. From Figure 3a it is clear that progressively more volumetric collapse occurred going from the self-supported, ∼70 μm^15^ to ∼300 nm and ∼30 nm films. At 700 °C, the ∼30 nm confined films collapsed approximately two times more (∼43%) as compared with the ∼300 nm films (∼22%). This large difference is also reflected by the much larger relative refractive indices for the ∼30 nm films. In addition, ∼30 nm films seem to have reached the maximum extent of volumetric collapse already at 700 °C as opposed to both ∼300 nm and ∼70 μm films. Figure 3a indicates a smooth trend of increasingly collapsed microporosity going from thick to thinner confined films such as those employed as selective layer of composite CMS membranes. A very similar trend has been reported in our recent work for PIM-1 and PTMSP ultrathin films,^36^ which may suggest that this behavior could be a general trend for amorphous microporous films. This thickness-dependent pyrolysis-induced densification may proceed in a fashion similar to physical aging which is known to accelerate in thinner films of other amorphous materials^17,23,37,38^ and has pronounced consequences for molecular fluid transport.^15,17,19,39,40^

TEM cross-sectional images for the ∼300 nm films pyrolyzed at the highest temperatures (700 and 800 °C), Figure 3b, seem to suggest that a slightly more heterogeneous CMS structure formed at 800 °C which may indicate some differences in the microporosity between the two samples. We note, however, that such an image analysis has an approximate character and it is difficult to draw conclusions about the changes in the total volume fraction of the micropores. Because of the fragility of pyrolyzed ∼30 nm films and reduced image contrast, TEM analysis in this case was very challenging and could only serve to confirm the approximate thickness range which was in agreement with ellipsometry (pristine film of 22 nm developed into 13.5 nm CMS film at 700 °C), Supporting Information, Figure S7.

Normalized Raman spectra for both film thickness ranges recorded for samples pyrolyzed at 700 and 800 °C are shown in Figure 3c. The spectra show “D” (for disordered) and “*G*” (for graphitic) peaks at ∼1320 and ∼1587 cm^–1^, respectively. Such peaks are typical for microcrystalline or amorphous carbonaceous materials. Consistent with ellipsometry, Figure 3a, the ∼300 nm films showed a significant change in the D/G ratio going from 700 to 800 °C. In amorphous carbons the development of a stronger “D” peak usually indicates increased ordering whereas the opposite is true for graphene.^41,42^ This suggests a more developed ordered structure for the ∼300 nm film at the higher pyrolysis temperature of 800 °C. On the other hand, the “*G*” peaks for both ∼30 nm films almost exactly overlap suggesting little difference in the CMS structure in the ultrathin films for the two highest pyrolysis temperatures. This finding is again consistent with ellipsometry data from Figure 3a indicating that these two thinner samples possessed nearly identical average refractive indices. The lower “*G*” peaks for the ∼30 nm CMS films as compared to ∼300 nm films indicate that the thinner samples possessed a more developed (ordered) CMS structure. This is supported by the peak area analysis shown in Table S1 in the Supporting Information. However, some degree of caution needs to be taken while interpreting D/G ratios because the exact trends will depend on many factors which are difficult to determine experimentally such as the crystallite size, stress, dopants, etc.^41^ As-recorded Raman spectra for samples pyrolyzed at 600 °C, shown in the Supporting Information, Figure S8, indicate that at 600 °C slightly more progression toward development of the CMS structure is again observed for the ∼30 nm films in comparison to the ∼300 nm films where only very weak “D” and “*G*” peaks are seen. Therefore, 600 °C clearly represents a transition regime where the precursor material has already lost its functional groups due to thermal decomposition^15^ but the turbostratic CMS structure only starts to develop. As demonstrated earlier,^11,15^ these transition CMS films possess particularly attractive gas separation properties often much above the gas permeability/selectivity trade-off lines representing state-of-the-art materials.

**Figure 4:**
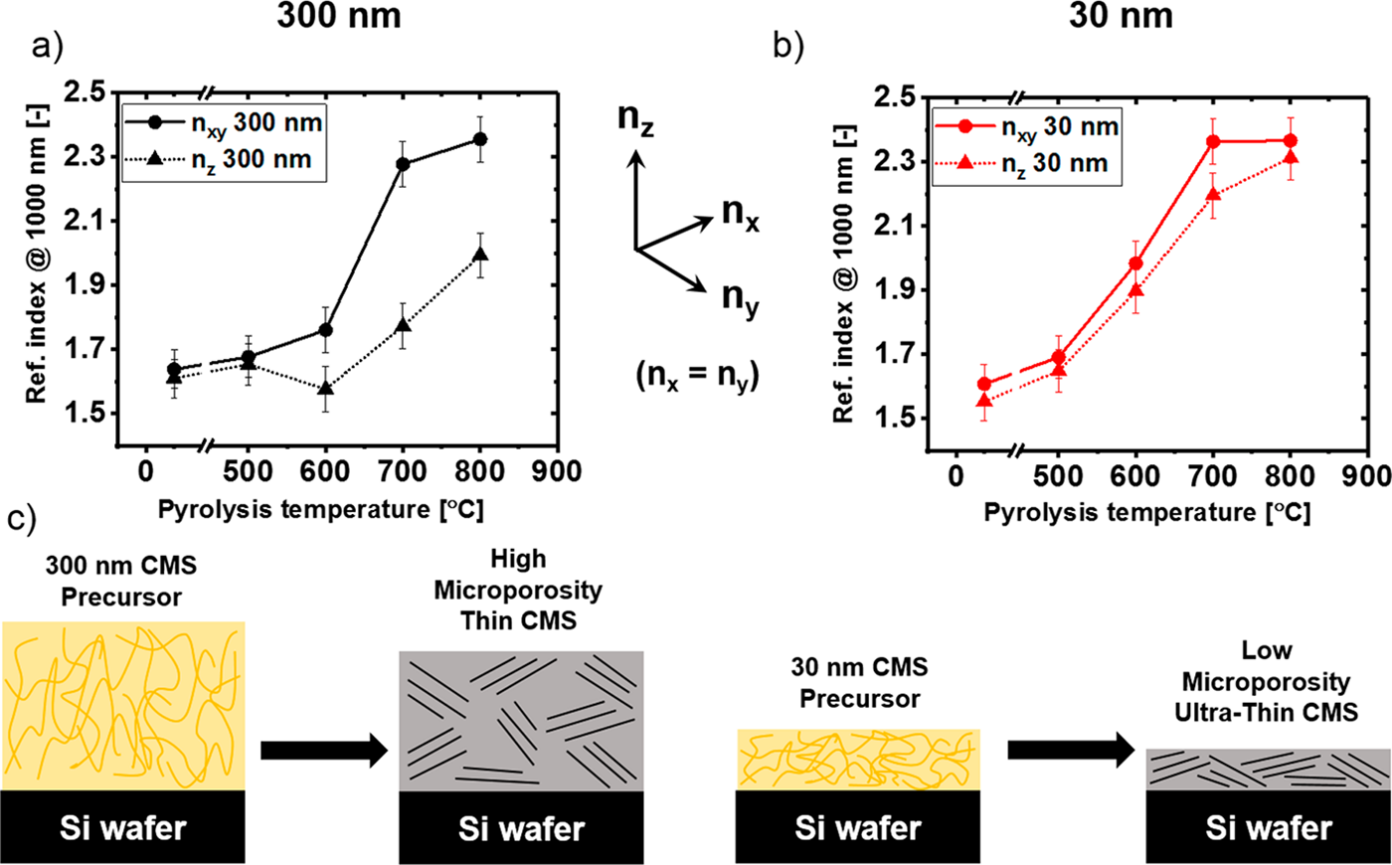
Results of the uniaxially anisotropic modeling of the pyrolytic collapse in (a) a thick (∼300 nm) and (b) thin (∼30 nm) films together with (c) a proposed scheme of the morphological transformations. Because of the lateral confinement to the substrate, the whole volume change as a result of pyrolysis is accommodated only by the change in film thickness.

To learn more about the morphology of the films during the pyrolytic collapse Figure 4 presents results of the uniaxial anisotropic modeling for both thick (a) and thin (b) films. In Figure 4a,b the in-plane (*n*_*xy*_) and out-of-plane (*n*_*z*_) components are presented which correspond to the refractive indices parallel and perpendicular to the silicon wafer substrate. We note here, that because of the confinement to the wafer substrate the lateral *x*-*y* dimensions of the films did not change during pyrolysis. Thickness change was thus identical to volume change, as opposed to free-standing CMS films which are known to shrink in all dimensions following pyrolysis. In the case of the thick films, both refractive index components seem to quickly separate above the pyrolysis temperature of 500 °C and the *n*_*z*_ component remains significantly lower than the *n*_*xy*_. This is in excellent agreement with our previous study^15^ as well as the behavior of much thicker supported films (10–70 μm)^30^ and suggests development of a pronounced preferential microscopic orientation of the forming graphene-like plates in the direction parallel to the substrate. This microscopic orientation is specific to supported film geometry and does not develop nearly as much in isotropic thick CMS films.^30^ The lower value of the *n*_*z*_ component in the thick films indicates that the oriented plates are to some extent separated horizontally by regions of lower density, and thus the thick films have higher interplane microporosity, Figure 4c. On the other hand, ∼30 nm films, Figure 4b, show much less separation between the *n*_*xy*_ and *n*_*z*_ components whereas the *n*_*xy*_ still remains slightly larger than *n*_*z*_. This remarkable development of thickness-dependent anisotropy corroborates a much more compacted structure of the thinner films, Figure 4c, which possess much less microporosity.

In addition, the presented results suggest that the lateral confinement to the substrate followed by the pyrolysis-induced volume (thickness) shrinkage are the primary drivers behind the structural orientations that develop within the thin CMS films. Similarly to our films produced by spin-coating, anisotropy (or birefringence) within pyrolysis-derived thin,^15^ as well as thick supported films,^30^ were observed for drop cast and slowly evaporated samples, respectively. Moreover, the lack of significant optical anisotropy in the pristine, nonpyrolyzed films further indicated that the structural orientation developed as a result of pyrolysis and was probably not triggered by the fabrication method of the precursor films.

**Figure 5:**
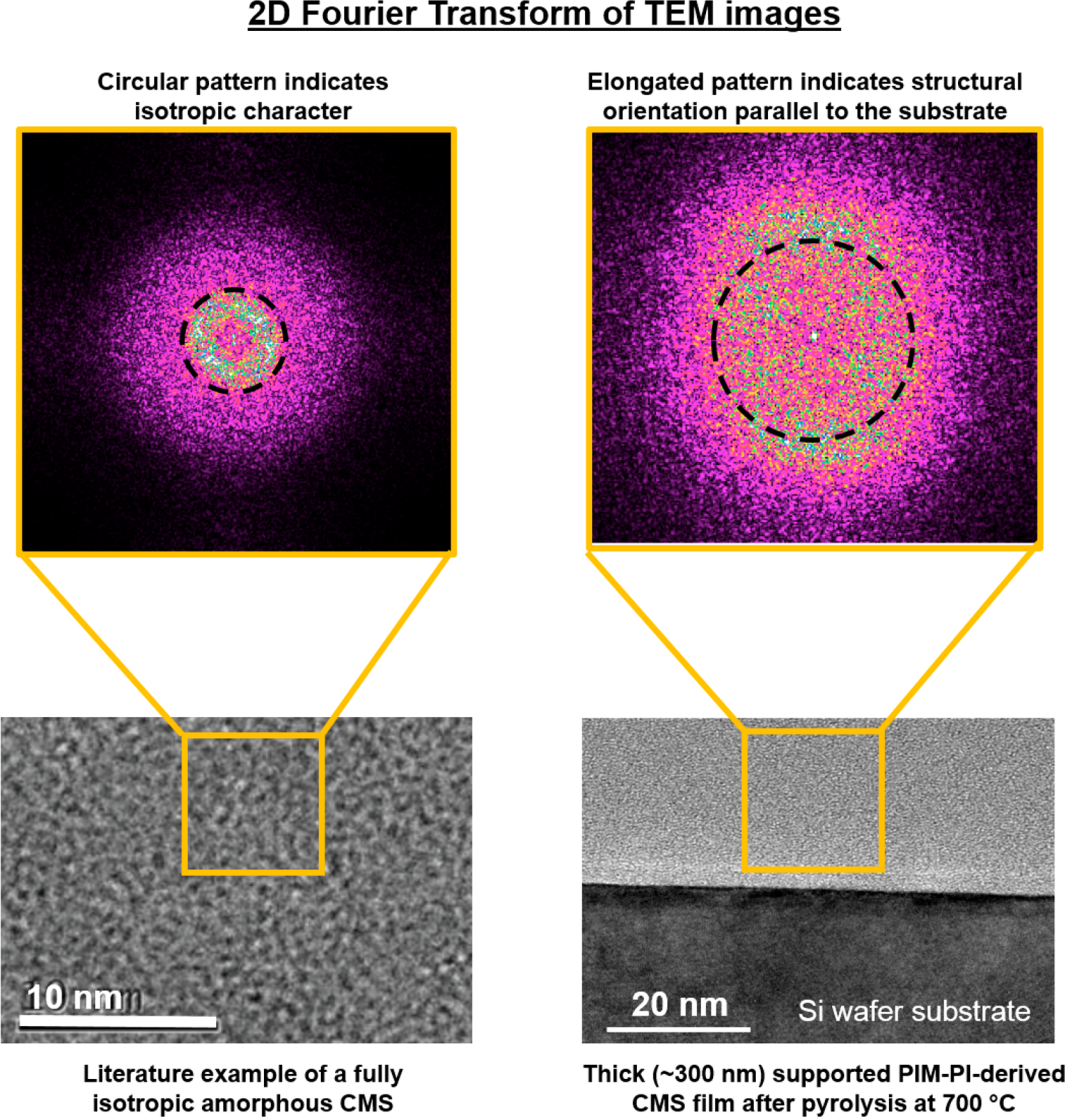
2D Fourier transforms for an isotropic amorphous CMS structure (left, adapted with permission from ref (43). Copyright 2013 Stefano Casciardi et al. under Creative Commons Attribution License.) and ∼300 nm Si-wafer supported PIM–PI derived CMS films from this study (right); dashed lines indicate perfect circles for reference.

Further suggestion for the existence of the internal orientation of in the supported CMS films is provided by the 2D Fourier transforms (2D FT) of the TEM images, Figure 5. While for a fully isotropic amorphous CMS structure (Figure 5, left) and in the absence of significant astigmatism the 2D FT pattern is expected to be perfectly circular, our ∼300 nm films show a slightly elongated ellipse (Figure 5, right). To account for the possible astigmatism of the imaging electron optics the 2D FT of the CMS film were evaluated in reference to the supporting Si wafer visible in the same image (identical imaging conditions) which could be assumed isotropic. The ellipticity of the CMS film pattern in the direction perpendicular to the supporting Si wafer was ∼7% greater than for the support (Supporting Information, Figure S9). This effect may suggest the slight preferential orientation within the TEM micrograph in parallel to the substrate which is in excellent agreement with the optical anisotropy detected by ellipsometry, Figure 4. However, we note that a more detailed study with appropriate isotropic control samples and careful correction for the possible imaging artifacts would have to be conducted to conclusively support TEM-based detection of preferential orientation within cross sections of the CMS thin films.

**Figure 6:**
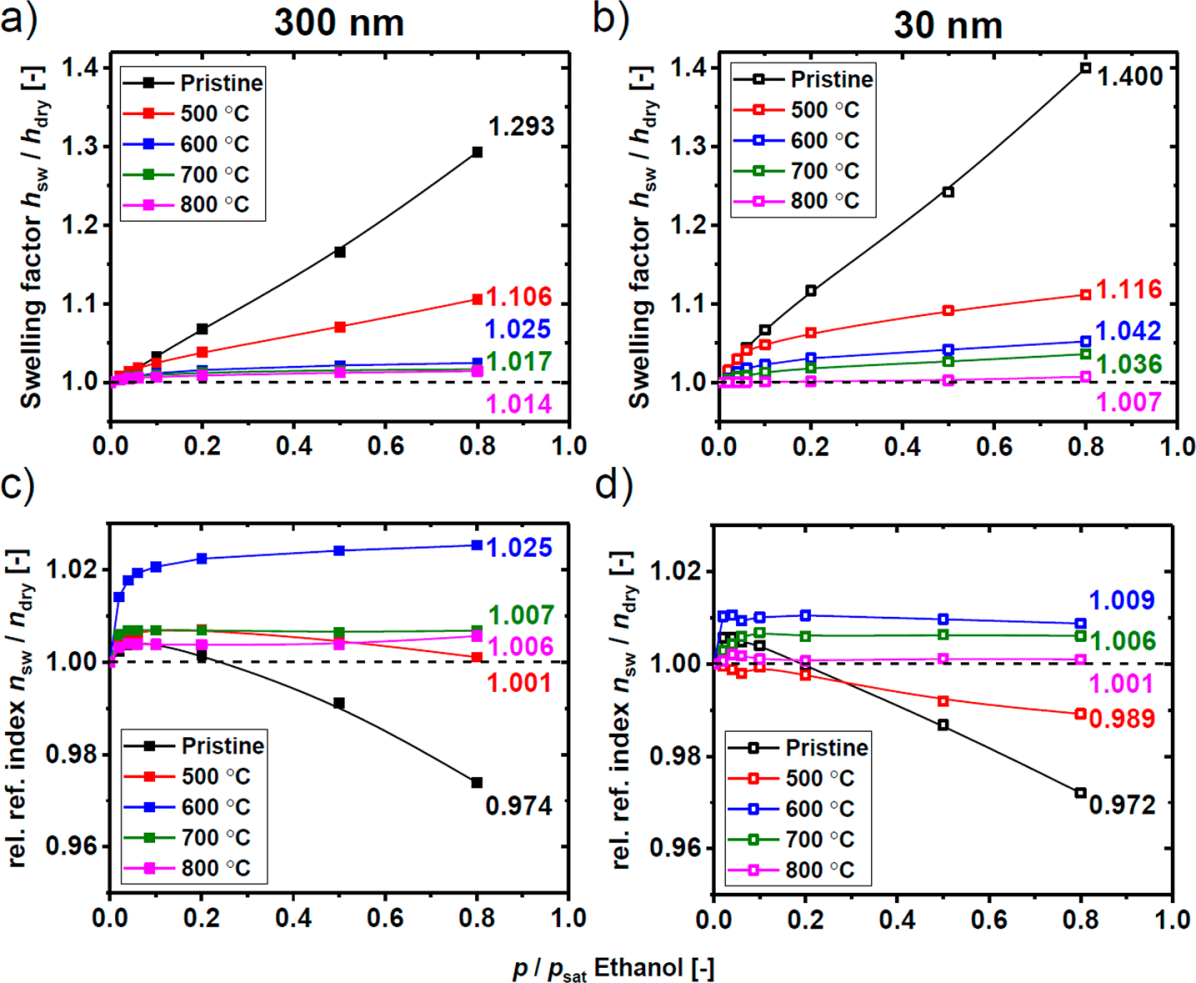
Swelling factors (a and b) and relative refractive indices (c and d) for the thick (∼300 nm) and thin (∼30 nm) pristine PIM–PI and carbon films upon exposure to ethanol vapors. Error bars are omitted for the sake of clarity; in each ease the errors are estimated at <5%.

The thickness-dependence in the developing microstructure may have significant consequences on the molecular fluid transport performance of the resulting CMS materials. This is because the larger micropore fractions generally lead to lower transport resistances and thus potentially more permeable membranes for gas separations. Gas sorption experiments (e.g., BET analysis) are a convenient way to elucidate the internal surface areas and porosities of polymeric and carbonaceous materials. However, such analyses remain very challenging in thin films (<several μm) because of the lack of necessary sensitivity. For the thin films in this study, we have used the particular advantage of in situ spectroscopic ellipsometry to independently study both the swelling and refractive index changes in pristine and carbon films as thin as ∼30 nm or less.^25^Figure 6 summarizes the obtained results by depicting the swelling factors (*h*_swollen_/*h*_dry_) and relative refractive indices (*n*_sw_/*n*_dry_) for both thick and thin films. The relative refractive indices were calculated from the average values of the *n*_*xy*_ and *n*_*z*_, as described earlier, and given at a wavelength of 1000 nm. For clarity of presentation and further discussion, we have included the numerical values at ethanol *p*/*p*_sat_ = 0.8 on each of the graphs in Figure 6.

The swelling data for freshly made CMS films using ethanol vapors as a model organic solvent (Figure 6a,b) reveal a very strong influence of the pyrolysis-induced physical and chemical transformations (e.g., loss of functional groups, π–π stacking, etc.) on the swelling behavior of the thin films with the maximum swelling factor rapidly decreasing with increasing pyrolysis temperature. The films pyrolyzed at the highest temperature of 800 °C hardly dilated in ethanol vapor atmosphere at all. The trends are very similar for the thick and thin films; however, the thin films dilated slightly more at each of the corresponding pyrolysis temperatures. This consistent difference between the thick and thin films will manifest itself later for the calculation of the penetrant concentration. The swelling isotherms were almost linear for the pristine PIM–PI precursor which was also observed in our earlier study on PIMs swelling in compressed CO_2_.^32^ In Figure 6c,d, a dashed line represents a relative refractive index of 1 to divide the two different regimes of the film density response to the sorbing penetrant. Whenever the relative refractive index rose above 1, the micropores of the material were filled with the penetrant. On the other hand, if the value reduced below 1, the swelling (molecular scale mixing and resulting volume dilation) dominated as the (optical) density of the film reduced below its initial value. This second effect occurred because the refractive index of liquid ethanol is much lower than the pristine or carbon films (*n*_ethanol_ = 1.36 vs *n*_pristine_ = 1.64) and thus a polymer (or carbon) with sorbed ethanol has a lower refractive index.^35^ In this view, the refractive index data (Figure 6c,d) provide corroboration of the results presented in Figure 4. While the differences in the relative refractive index behavior of the thick and thin films of the pristine materials were small, the pyrolyzed films (500–800 °C) showed a significant thickness dependence. In particular, at 500 °C the relative refractive index immediately reduced below 1, whereas for the thick film it increased, going through a maximum and reduced at higher ethanol vapor pressures. This effect indicated that, while both thick and thin films were still largely polymeric at the beginning of the pyrolytic decomposition (degradation temperature onset for bulk film is ∼520 °C), the microporosity in the thinner film was already very significantly reduced. Ethanol was forced to swell the matrix resulting in a progressively decreasing relative refractive index and increased swelling. This effect amplified at 600 °C where the increase of the relative refractive index in the thinner film was only about a third of that for the thicker film and, correspondingly, the maximum swelling was much larger (1.042 versus 1.025). The samples pyrolyzed at 700 °C continued the same trend while at the same time showing a much smaller response of the relative refractive index which is consistent with the reduced microporosity accessible to ethanol as a result of further progressing pyrolytic collapse. The thick film pyrolyzed at 800 °C showed very little swelling (swelling factor = 1.014) but displayed a microporosity filling effect as evidenced by the similar relative refractive index to that of the 700 °C film. In the thinner film pyrolyzed at 800 °C virtually no response was observed either in swelling or in the refractive index. For this sample, apparently the structure compacted so significantly that ethanol was not able either to enter the micropores or sorb within the matrix causing swelling.

**Figure 7:**
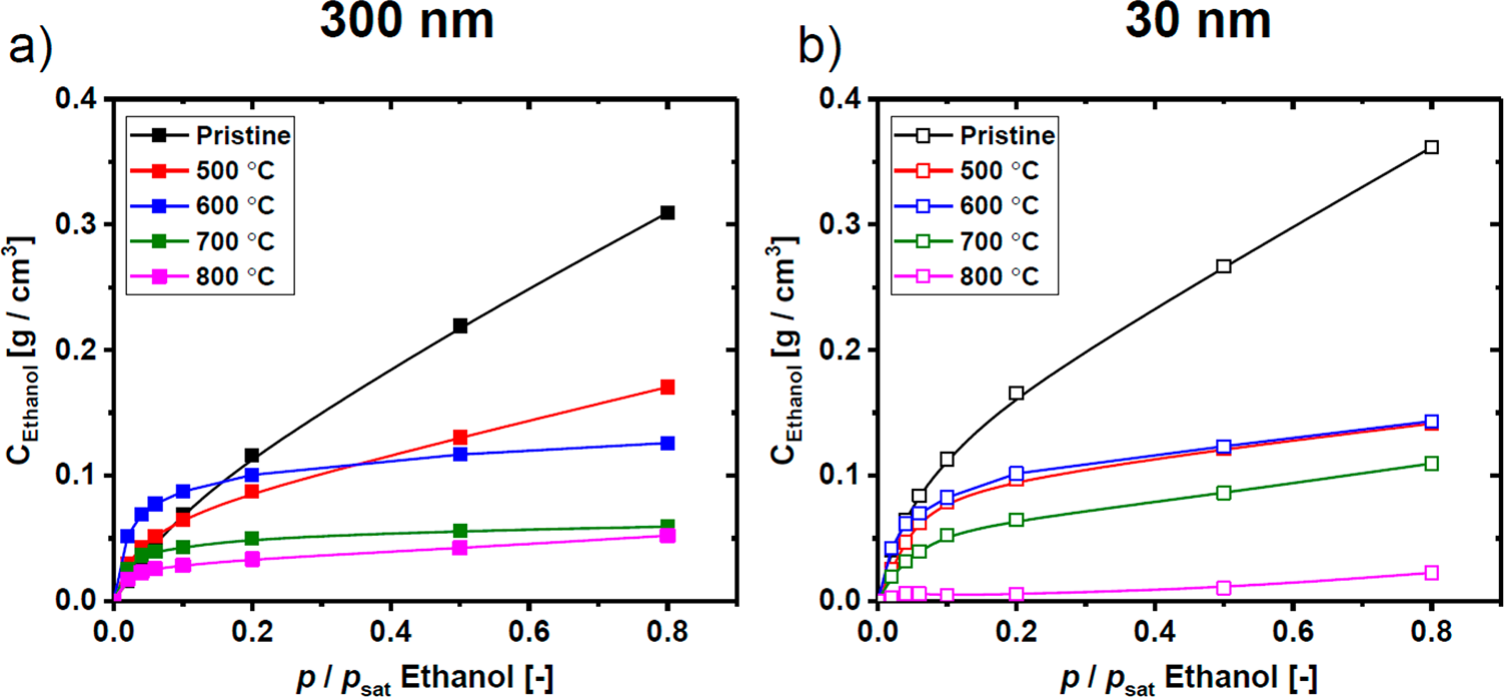
Concentration of ethanol estimated via the Clausius-Mossotti calculations for (a) thick (∼300 nm) and (b) thin (∼30 nm) pristine PIM–PI and carbon films (500, 600, 700, and 800 °C). Error bars are omitted for the sake of clarity; in each ease the errors are estimated at <5%.

The information about film swelling and refractive index can be converted into estimated penetrant concentrations using the well-known Clausius-Mossotti approach which has been previously utilized in similar ellipsometry studies.^32,44,45^ The results for all studied samples are shown in Figure 7 and clearly indicate that, in general, pyrolysis leads to a reduction of the penetrant sorption with higher pyrolysis temperatures reducing sorption even further. This behavior further indicates that the pyrolysis-induced collapse dominates over microporosity creation accompanying the chemical transformations. For the pristine PIM–PI precursor, the larger swelling of the thinner film dominated and produced slightly larger estimated ethanol concentrations. We suggest this may be related with an increased effect of the more swellable free surface at the polymer/vapor interface.^20,25^ For the samples pyrolyzed in the transitional temperature range of 500 and 600 °C, despite significant differences between the thick and thin films, as shown in Figure 6, the estimated concentrations did not significantly differ. Apparently, the loss of microporosity related with reducing film thickness from 300 to 30 nm was compensated by the significantly larger swelling of the thinner films in each pair which, as a result, produced similar ethanol concentration. At 700 °C the swelling of the thinner film, which was almost two times larger than for the thicker film, seems to dominate and resulted in slightly larger concentrations. At 800 °C, as discussed earlier, some accessible microporosity of the thicker film allowed ethanol to sorb into the structure, which was not the case in the already almost completely impenetrable thinner film pyrolyzed at the same temperature.

This work may serve to provide more direct evidence for some of the anomalies in transport behavior of membranes based on thin amorphous selective CMS and PIM–PI films. In particular, slower than expected fluid transport by a simple extrapolation from the bulk properties of thick films^15,39,46^ may have its origin in the significantly compacted microporous structure in thinner layers. To avoid those hurdles excessive reduction of the selective layer thickness beyond several hundred nanometers needs to be avoided unless the collapse of the internal microporosity could be avoided by other means.

## 4 Conclusions

Pyrolytic collapse and ethanol-induced swelling in a thin polyimide of intrinsic microporosity (PIM–PI) precursor and derived carbon molecular sieve (CMS) films in two thickness ranges, ∼300 nm and ∼30 nm, were investigated using interference-enhanced spectroscopic ellipsometry, transmission electron microscopy and Raman spectroscopy. We discovered that pyrolysis of thin precursor films led to significant orientation of the CMS structure in the direction parallel to the substrate guided by the lateral constraint. A strong thickness dependence of the degree of volumetric collapse was found: ultrathin films collapsed to a much larger extent and became significantly denser than their thicker counterparts. Raman spectroscopy suggested that the ultrathin films in the range of ∼30 nm developed the CMS structure earlier (i.e., at lower pyrolysis temperatures) than the thicker ∼300 nm films. In both thicker and thinner films, the degree of ethanol-induced swelling strongly decreased with increasing pyrolysis temperature. For the thinner ∼30 nm films, in the transition carbon region (500–600 °C), lower extent of pore filling by the sorbing ethanol vapor was found as a result of the reduced microporosity. This effect was offset by a slightly larger swelling leading ultimately to similar calculated ethanol concentration for the thicker and thinner films. At higher pyrolysis temperatures, the differences between the thicker and thinner films amplified and at 800 °C the thinner film became virtually impenetrable to the ethanol vapor, in contrast to the thicker film pyrolyzed at the same temperature.

The results presented in this work are anticipated to improve the understanding of the thickness-dependent structural development in thin-film CMS membranes, as well as their interaction with technologically important separations with feeds involving condensable penetrants such as CO_2_, organic solvents, or olefins and paraffins. Our results reinforce previous findings that excessive reduction of the selective layer thickness in amorphous microporous materials (such as PIMs or CMS) beyond several hundred nanometers may lead to extremely compact structures which in turn may not be optimal for molecular transport and separation.

## Supporting Information Available

Thermogravimetric, volumetric, and density data for bulk powder and ∼70 μm films of the CMS precursor PIM-polyimide SBFDA-DMN (in comparison to another non-PIM polyimide); optical model fitting; kinetic data for an in situ ethanol swelling experiment; relative refractive index plotted as a function of swelling factor of thick and thin films; swelling factor plotted versus ethanol concentration; atomic force microscopy images; optical images; cross-sectional transmission electron microscopy images; and Raman spectra for both thick and thin films (PDF)
