# Supplementary material for: Nano-Confinement Effects on Structural Development and Organic Solvent-Induced Swelling of Ultrathin Carbon Molecular Sieve Films
Source: ACS Appl Mater Interfaces. 2021 Apr 28;13(18):21765–74. doi: 10.1021/acsami.1c03392 (PMC8289180; doi:10.1021/acsami.1c03392)
Supplement: Supplementary file 1 — am1c03392_si_001.pdf [file am1c03392_si_001.pdf]

Supporting Information

Nano-Confinement Effects on Structural Development and Organic Solvent-Induced Swelling of Ultra-Thin Carbon Molecular Sieve Films

Wojciech Ogieglo[1], Kepeng Song[2], Cailing Chen[2], Qiong Lei[2], Yu Han[2], Ingo Pinnau[1,*]

[1] Functional Polymer Membranes Group, Advanced Membranes and Porous Materials Center, Division of Physical Sciences and Engineering, King Abdullah University of Science and Technology, 23955 Thuwal, Saudi Arabia

[2] Nanostructured Functional Materials, Advanced Membranes and Porous Materials Center, Division of Physical Sciences and Engineering, King Abdullah University of Science and Technology, 23955 Thuwal, Saudi Arabia

*ingo.pinnau@kaust.edu.sa

Table of contents:

Figure S1. Powder and thick film TGA-derived weight loss and the relative volume, relative mass and relative density plotted against the pyrolysis temperature for bulk (thick films) of the PIM and non-PIM polyimides.

Figure S2. An example of ellipsometric analysis including the scheme of the optical model, fitted parameters (**bold**), and the resulting spectral fits.

Figure S3. In-situ swelling of an initially ~300 nm precursor film pyrolyzed at 600 °C (resulting in ~239 nm carbon molecular sieve film) exposed to increasing ethanol vapor pressures.

Figure S4. Optical and atomic force microscopy images of the ~30 nm pristine and CMS films.

Figure S5. Optical and atomic force microscopy images of the ~300 nm pristine and CMS films.

Figure S6. Swelling factors ($h_{swollen}$ / h_{dry}) plotted versus ethanol concentration for all analyzed thick and thin pristine and CMS films.

Figure S7. Transmission electron microscopy images of the thick films pyrolyzed at 700 and 800 °C showing a larger area and the interface with the silicon wafer substrate.

Figure S8. 2D Fourier Transformation of the TEM images of the CMS film and the supporting Si wafer.

Figure S9. As-recorded Raman spectra for the ~30 and ~300 nm CMS films.

Figure S10. Peak analysis of the Raman spectra.

Table S1. "D" and "G" peak area ratios derived from Lorentzian fitting of the normalized Raman spectra from Figure 2c. In figures below, the details of the Peak Analysis are presented.

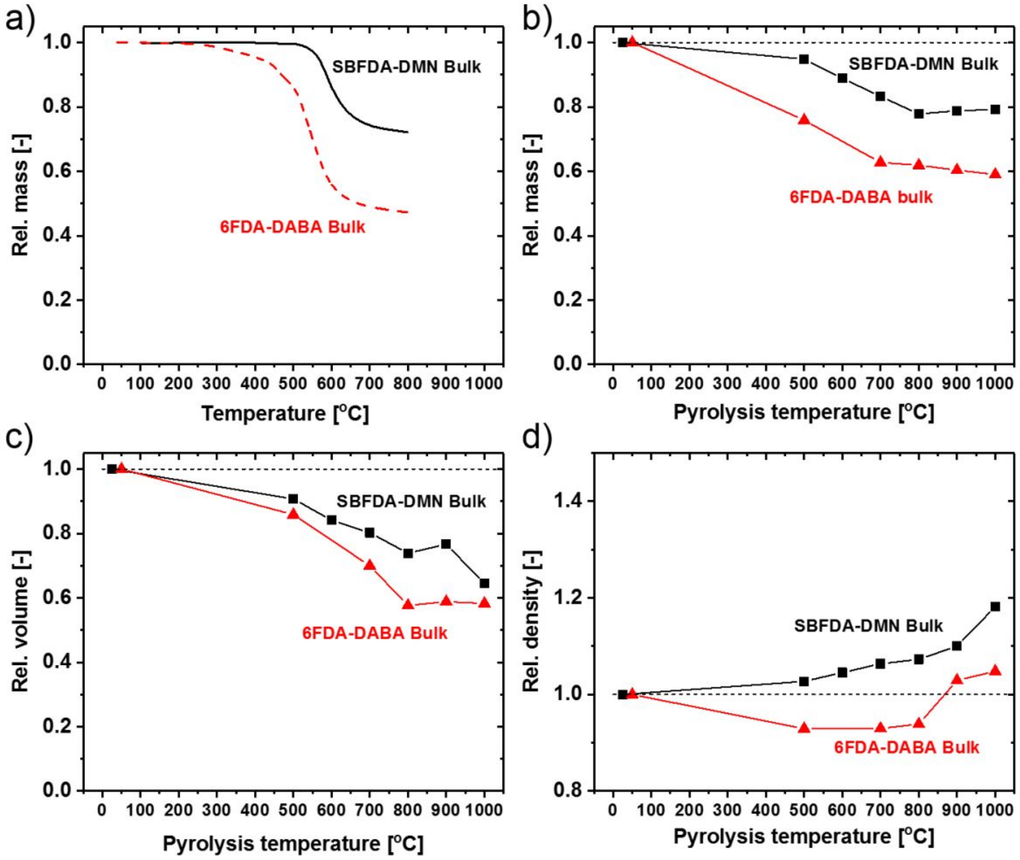

Figure S1. Powder (a) and thick film (b) TGA-derived weight loss as well as thick film relative volume (c) and relative density (d) plotted against the pyrolysis temperature for bulk (thick films) of the PIM and non-PIM polyimides. “Bulk” refers to self-supported ~70 µm films.

Sample: CSP_03 700C drying n2 before ethanol run

Fit Results

MSE = 4.954
Thickness # 3 = 240.15 ± 0.354 nm
E Inf = 0.303 ± 0.2147
IR Amp = 0.452 ± 0.0712
dZ_A = -0.505261 ± 0.0031659
n_o of Biaxial @ 1000.0 nm = 2.27744
n_e of Biaxial @ 1000.0 nm = 1.77218

Optical Model

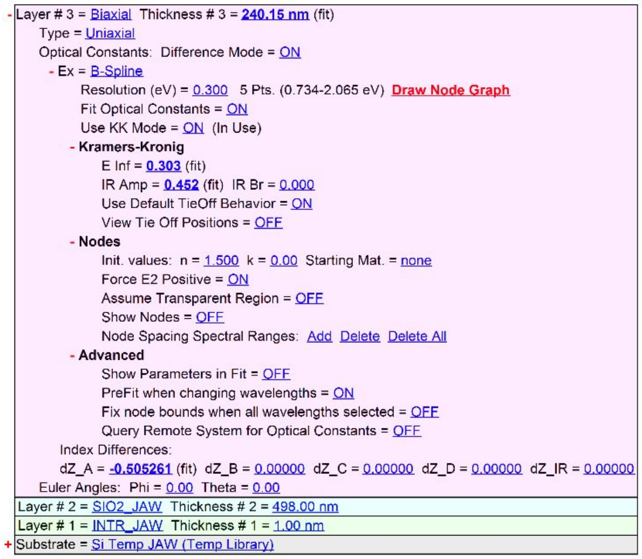

Experimental and Model Generated Data Fits

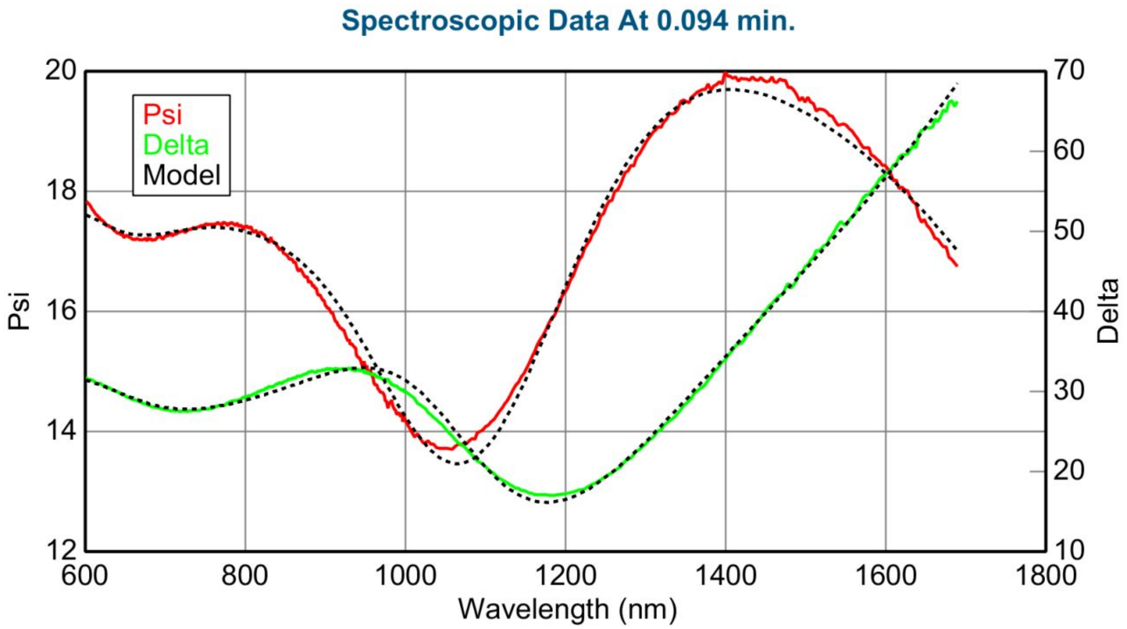

Figure S2. An example of ellipsometric analysis including the scheme of the optical model, fitted parameters (**bold**), and the resulting spectral fits.

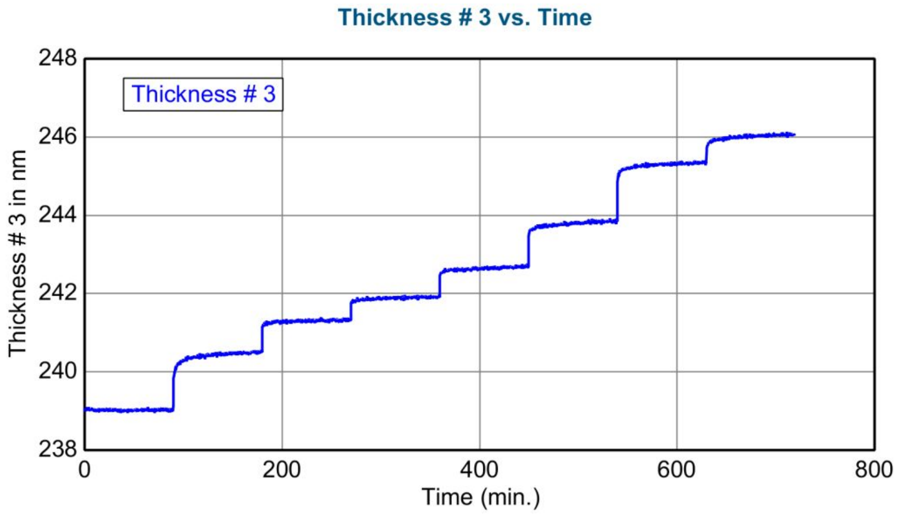

Figure S3. In-situ swelling of an initially ~300 nm precursor film pyrolyzed at 600 °C (resulting in ~239 nm carbon molecular sieve film) exposed to increasing ethanol vapor pressures.

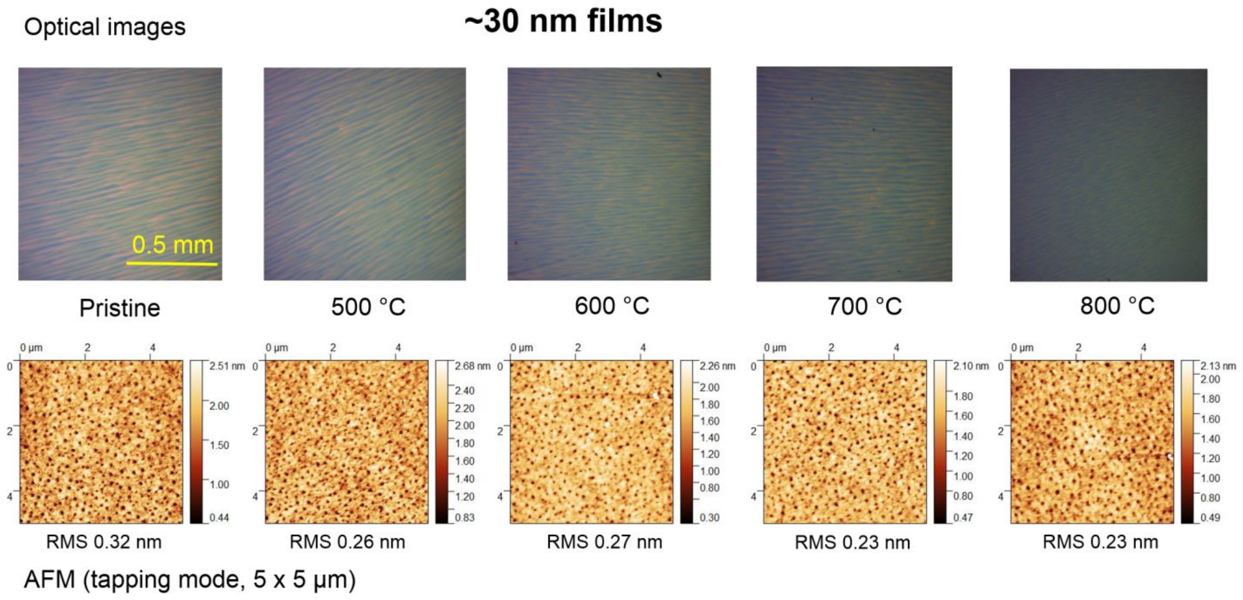

Figure S4. Optical and atomic force microscopy images of the ~30 nm pristine and CMS films.

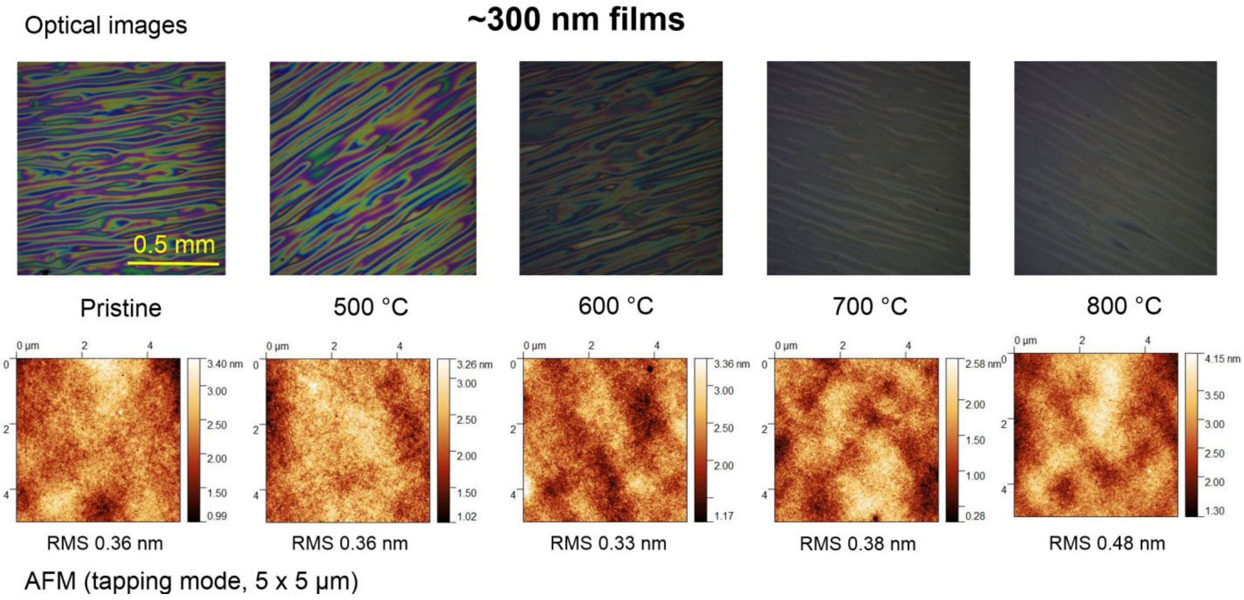

Figure S5. Optical and atomic force microscopy images of the ~300 nm pristine and CMS films.

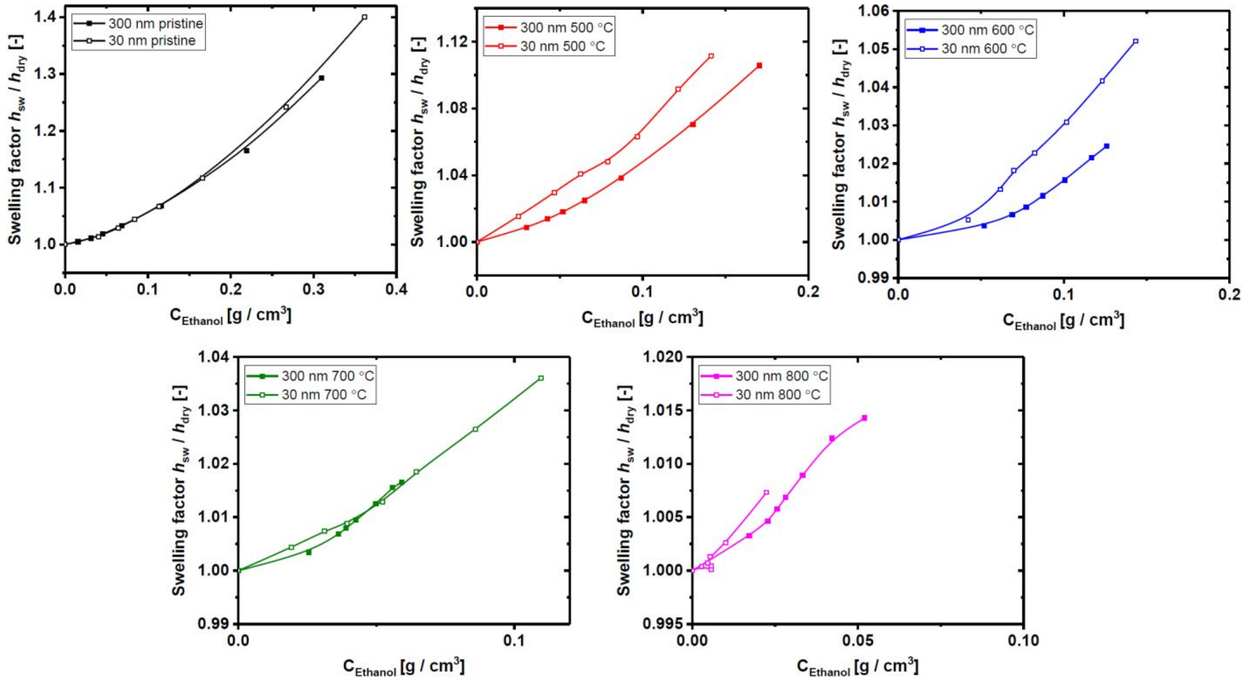

Figure S6. Swelling factors plotted versus ethanol concentration for all analyzed thick and thin pristine PIM-PI and CMS films.

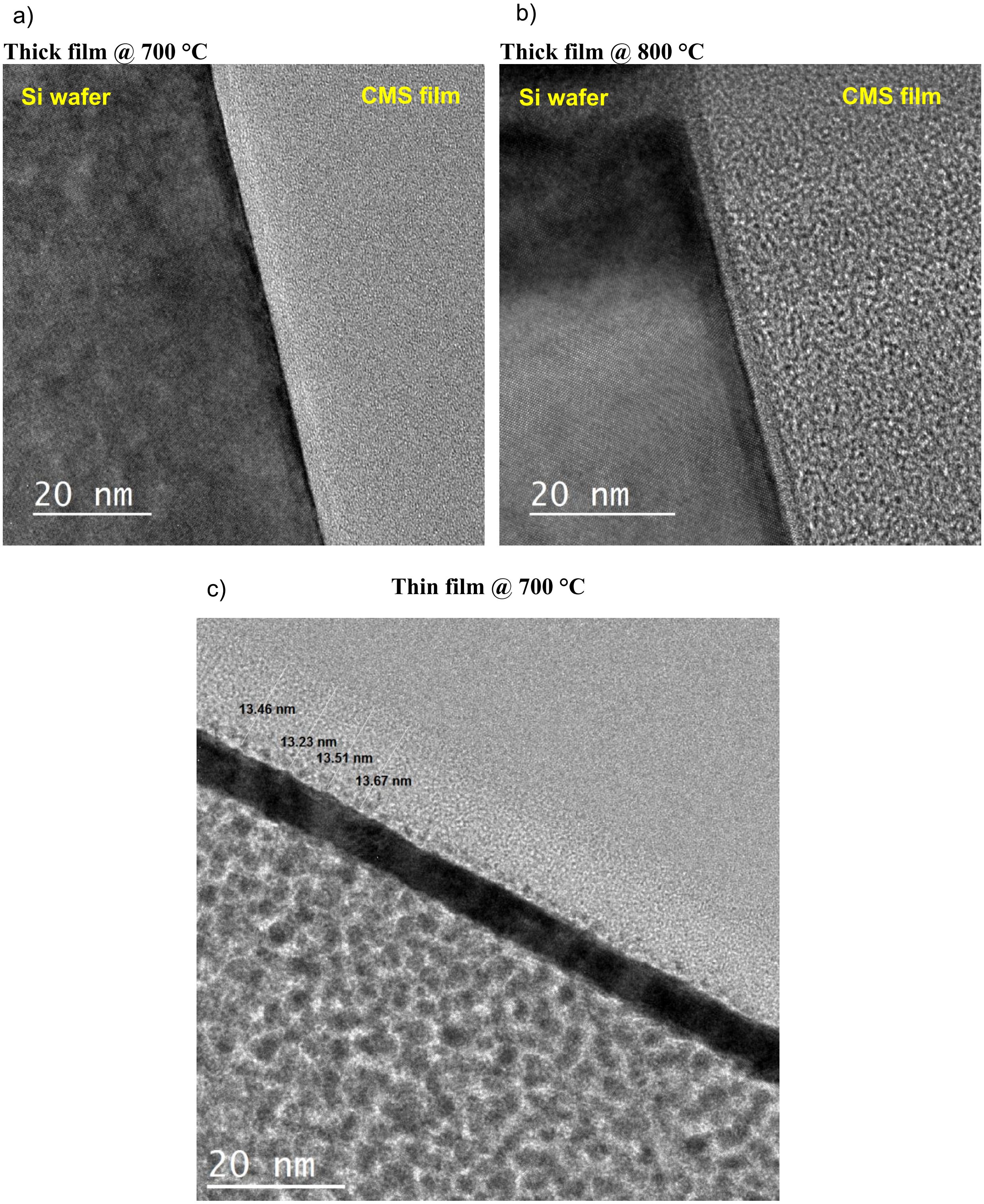

Figure S7. Transmission electron microscopy cross-sectional images of the thick films pyrolyzed at (a) 700 °C and (b) 800 °C showing a larger area and the interface with the silicon wafer substrate. For the thin film (c) an approximate thickness of the pyrolyzed film can be detected.

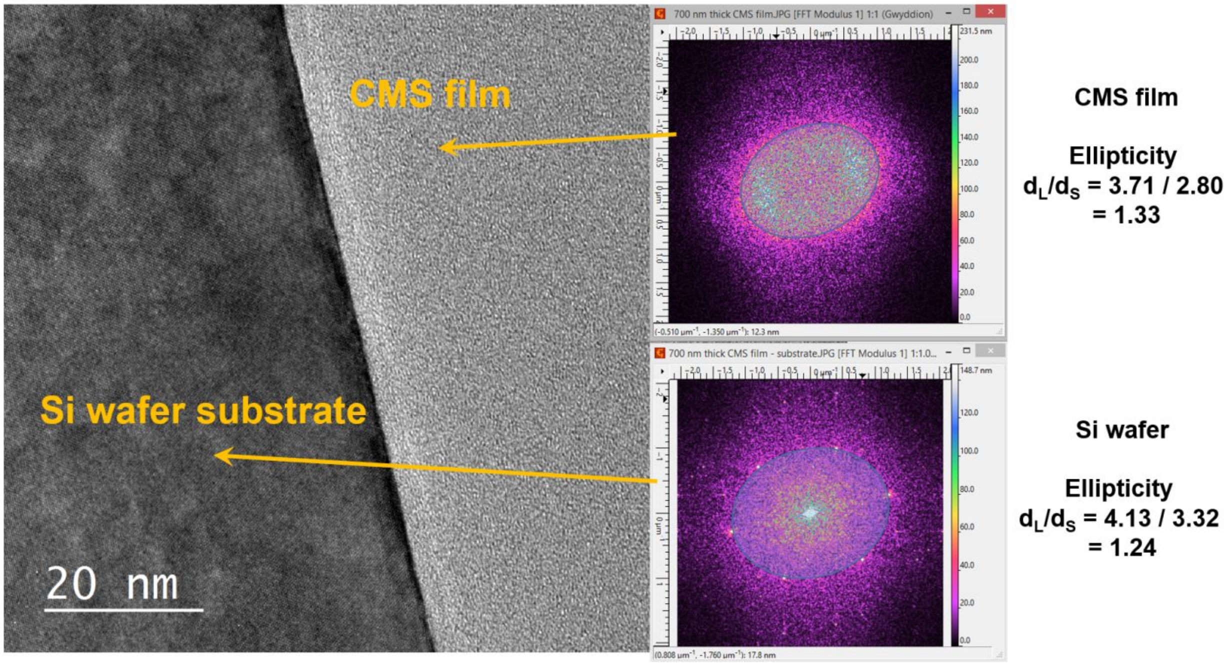

Figure S8. 2D Fourier Transformation (2D FT) of the TEM image for the CMS film as well as the supporting Si wafer structure. The slightly larger ellipticity of the CMS film 2D FT pattern relative to the ellipticity of the Si wafer substrate is interpreted as a possibility of a slight structural orientation of within the CMS films parallel to the substrate.

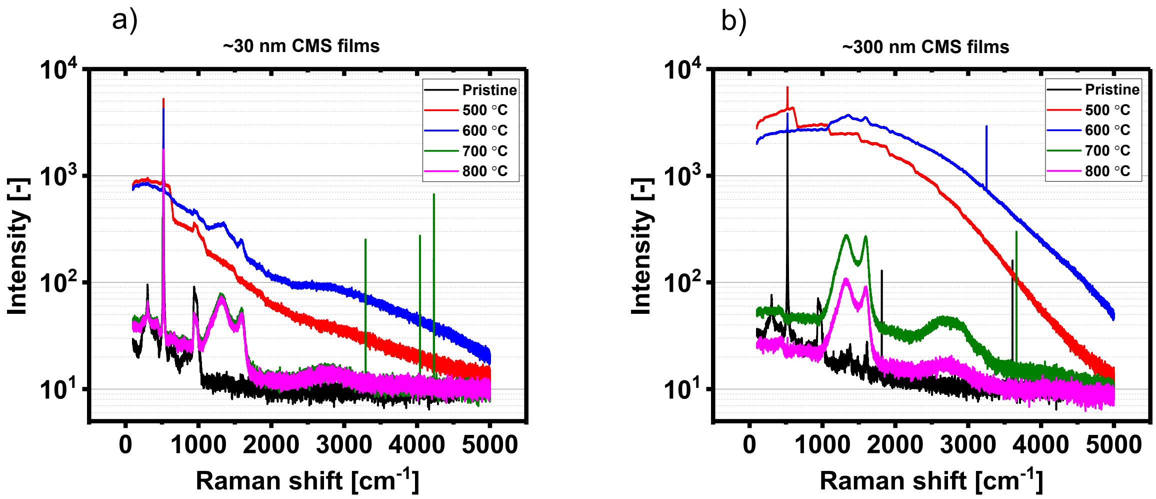

Figure S9. As-recorded Raman spectra for the (a) ~30 and (b) ~300 nm CMS films.

Table S1. "D" and "G" peak area ratios derived from Lorentzian fitting of the normalized Raman spectra from Figure 2c. In figures below, the details of the Peak Analysis are presented.

**Sample**	***A*(D)/*A*(G)**
~30 nm 700 °C	4.05
~30 nm 800 °C	4.04
~300 nm 700 °C	3.05
~300 nm 800 °C	3.36

a)

Peak Analysis

Data Set:[Book2]Sheet1!K"Normalized 30 nm 700C" Date:02/09/2019
BaseLine:Line
Chi^2=1.65708E-003 Adj. R-Square=9.71213E-001 # of Data Points=2253
SS=3.72180E+000 Degree of Freedom=2246

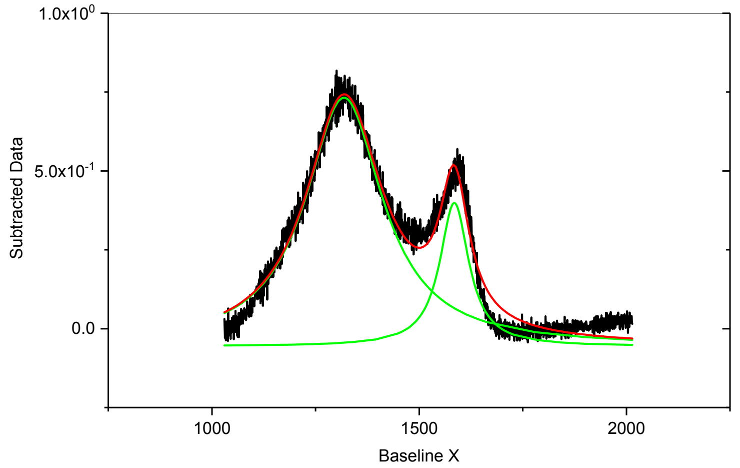

Fitting Results

Peak Index	Peak Type	Area Intg	FWHM	Max Height	Center Grvty	Area IntgP
1	Lorentz	247.05791	246.44991	0.80798	1318.5777	81.67767
2	Lorentz	55.42123	83.69975	0.45249	1587.51934	18.32233

b)

Peak Analysis

Data Set:[Book2]Sheet1!L"Normalized 30 nm 800C" Date:02/09/2019
BaseLine:Line
Chi^2=1.39450E-003 Adj. R-Square=9.75051E-001 # of Data Points=1888
SS=2.62306E+000 Degree of Freedom=1881

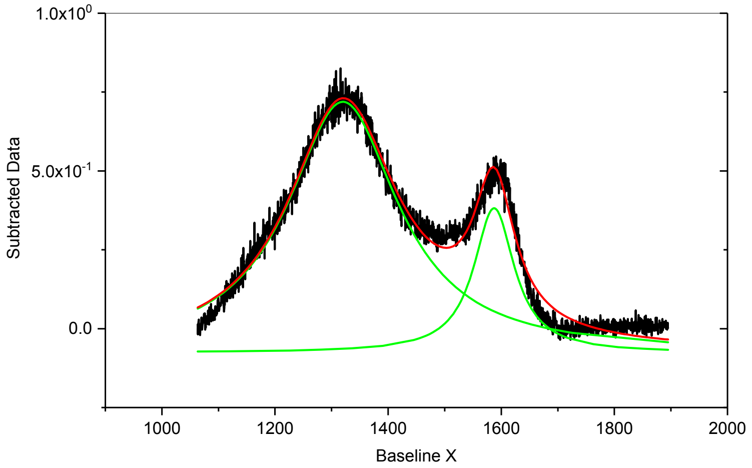

Fitting Results

Peak Index	Peak Type	Area Intg	FWHM	Max Height	Center Grvty	Area IntgP
1	Lorentz	236.11689	237.07288	0.79491	1320.03438	80.1809
2	Lorentz	58.36333	87.45418	0.45754	1587.32546	19.8191

c)

Peak Analysis

Data Set:[Book1]Sheet1!K"Normalized 300 nm 700C" Date:02/09/2019

BaseLine:Line

Chi^2=1.43368E-003 Adj. R-Square=9.85172E-001 # of Data Points=2336

SS=3.33905E+000 Degree of Freedom=2329

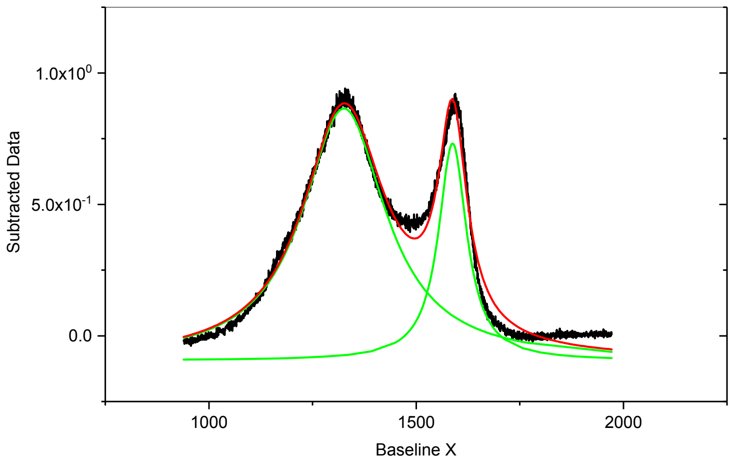

Fitting Results

Peak Index	Peak Type	Area Intg	FWHM	Max Height	Center Grvty	Area IntgP
1	Lorentz	309.40022	243.61542	0.95842	1325.84798	75.28513
2	Lorentz	101.57101	82.83774	0.82559	1587.92548	24.71487

d)

Peak Analysis

Data Set:[Book1]Sheet1!L"Normalized 300 nm 800C" Date:02/09/2019

BaseLine:Line

Chi^2=1.48244E-003 Adj. R-Square=9.80876E-001 # of Data Points=2696

SS=3.98627E+000 Degree of Freedom=2689

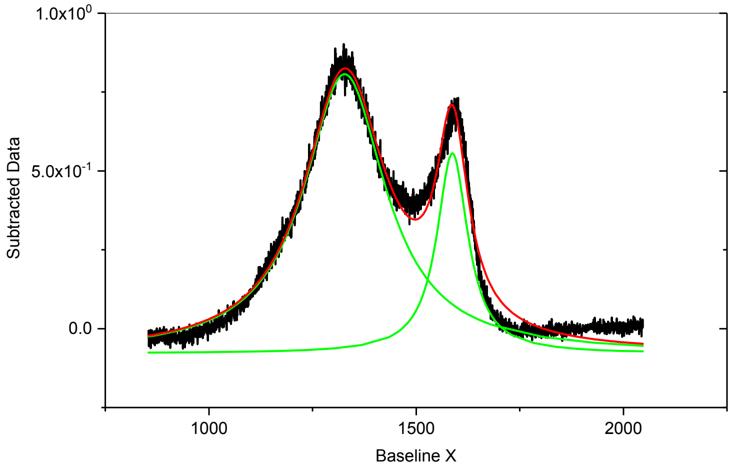

Fitting Results

Peak Index	Peak Type	Area Intg	FWHM	Max Height	Center Grvty	Area IntgP
1	Lorentz	289.51079	239.82278	0.88491	1327.50258	77.05825
2	Lorentz	86.19302	91.22531	0.634	1587.40545	22.94175

Figure S10. Peak analysis of the Raman spectra for ~30 thin CMS films pyrolyzed at 700 and 800 °C (a, b) and ~300 nm CMS films pyrolyzed at 700 and 800 °C (c, d).
